# Free radical scavenger, edaravone, reduces the lesion size of lacunar infarction in human brain ischemic stroke

**DOI:** 10.1186/1471-2377-11-39

**Published:** 2011-03-30

**Authors:** Taizen Nakase, Shotaroh Yoshioka, Akifumi Suzuki

**Affiliations:** 1 Department of Stroke Science, Research Institute for Brain and Blood Vessels - Akita 6-10 Sensyu Kubota Machi, Akita, 010-0874, Japan

## Abstract

**Background:**

Although free radicals have been reported to play a role in the expansion of ischemic brain lesions, the effect of free radical scavengers is still under debate. In this study, the temporal profile of ischemic stroke lesion sizes was assessed for more than one year to evaluate the effect of edaravone which might reduce ischemic damage.

**Methods:**

We sequentially enrolled acute ischemic stroke patients, who admitted between April 2003 and March 2004, into the edaravone(-) group (n = 83) and, who admitted between April 2004 and March 2005, into the edaravone(+) group (n = 93). Because, edaravone has been used as the standard treatment after April 2004 in our hospital. To assess the temporal profile of the stroke lesion size, the ratio of the area [T2-weighted magnetic resonance images (T2WI)/iffusion-weighted magnetic resonance images (DWI)] were calculated. Observations on T2WI were continued beyond one year, and observational times were classified into subacute (1-2 months after the onset), early chronic (3-6 month), late chronic (7-12 months) and old (≥13 months) stages. Neurological deficits were assessed by the National Institutes of Health Stroke Scale upon admission and at discharge and by the modified Rankin Scale at 1 year following stroke onset.

**Results:**

Stroke lesion size was significantly attenuated in the edaravone(+) group compared with the edaravone(-) group in the period of early and late chronic observational stages. However, this reduction in lesion size was significant within a year and only for the small-vessel occlusion stroke patients treated with edaravone. Moreover, patients with small-vessel occlusion strokes that were treated with edaravone showed significant neurological improvement during their hospital stay, although there were no significant differences in outcome one year after the stroke.

**Conclusion:**

Edaravone treatment reduced the volume of the infarct and improved neurological deficits during the subacute period, especially in the small-vessel occlusion strokes.

## Background

Acute treatment of ischemic stroke requires a quick response after the onset of the injury; for example, thrombolytic therapy using recombinant tissue-type plasminogen activator was effective when applied within 3 hours of onset to be effective in US and Japan [1], and 4.5 hrs in Europe [2,3]. Inflammatory responses and injury to the endothelia of blood vessels have been simultaneously observed in acute ischemic lesions [4,5]. Therefore, it is critical not only to restore blood flow but also to protect neurons in order to reduce brain damage following an ischemic insult. Moreover, an extension of the therapeutic window would be beneficial to treat a broader range of patients with acute brain ischemia [6,7].

A somewhat novel line of therapy used to treat ischemic stroke is the use of free radical quenching agents [8]. Edaravone, a free radical scavenger, has been clinically available in Japan since 2001 and has been reported to improve clinical outcomes in patients exhibiting ischemic strokes [9]. Experimental studies have revealed that the possible mechanisms of edaravone are decreasing oxidative stress [10], protecting neurovascular units [11,12], and reducing the activation of microglia [13] after ischemic stress. Since most published studies have focused on the effect of edaravone during the acute therapeutic stages of cerebral ischemia, it is unclear if edaravone is effective in the chronic stages of cerebral ischemia. Therefore, we set out to determine if there are any changes in lesion size with edaravone treatment during the acute and chronic phases following cerebral ischemia and to explore whether the expansion of ischemic lesion was dependent on a free radical scavenging.

## Methods

### Patients

The use of edaravone for the treatment of cerebral ischemia at our hospital was extremely limited prior to March 2004. Upon approval of its use by the Medical Ethics Committee in April 2004, edaravone was readily available as a therapeutic agent against cerebral strokes. This study designed to examine the use of edaravone and the outcomes following stroke was approved by the Medical Ethics Committee in Research Institute for Brain & Blood Vessels -Akita. Patients admitted to the hospital within 24 hs after onset with a diagnosis of acute ischemic stroke between April 2003 and March 2004 were treated with standard medications without edaravone, and these patients were classified into an edaravone-untreated group [edaravone(-) group; n = 83]. Similarly, patients admitted between April 2004 and March 2005 were treated with standard medications in addition to an edaravone intravenous drip infusion (Radicut^®^, Mitsubishi Tanabe Pharma Corporation, Tokyo, Japan: 30 mg twice a day, which was the dosage approved by Japanese Ministry of Health, Labour and Welfare), and these patients were classified into an edaravone-treated group [edaravone(+) group; n = 93]. All patients were clinically diagnosed with ischemic stroke, which was confirmed by magnetic resonance imaging (MRI: Sigma1.5T, GE Medical Systems) on admission. Transverse T2-weighted images (T2WI, TR: 3600 sec, TE: 96 sec) and diffusion-weighted images (DWI, TR: 5800 sec, TE: 76.2 sec) were acquired with a slice thickness of 5 mm. Magnetic resonance angiography (MRA) was performed using the three-dimensional time-of-flight method, and images were rendered using the maximum intensity projection method. All patients were categorized into four groups according to the classification of their stroke subtype by the Trial of Org 10172 in Acute Stroke Treatment (TOAST) [14]: cardioembolism, large-artery atherosclerosis, small-vessel occlusion and others. Ischemic stroke patients with unknown etiology were excluded from this study.

The ischemic lesion at onset was observed based on the findings of the DWI-positive area which was confirmed by the apparent diffusion coefficient (ADC) map. The lesion in the later phases was assessed by the findings of the T2WI-positive area. The maximum lesion size in a single slice was adapted to the calculation according to a previously reported method [15]. The measurement of the slices was performed by the assistance of a computer software program (Synapse 3.1.1, Fujifilm Medical Systems USA Inc.). To evaluate the temporal profile of the stroke lesion, the ratio of the lesion size at a later period against the onset lesion size (T2WI/DWI) was calculated. The average ratio was assessed several times after the onset: subacute period (1-2 months), early chronic period (3-6 months), late chronic (7-12 months) and old (≥13 months) periods.

In addition to infarct sizes, several other factors were compared between the edaravone(+) and edaravone(-) groups. The National Institutes of Health Stroke Scale (NIHSS) was used to evaluate the neurological deficits of the patients upon admission for the cerebral stroke as well as at the time of discharge. The duration of in-hospital treatment was also analyzed. The activity of daily living (ADL) level was assessed at 1 year following stroke onset based on patients' clinical records and evaluated using the modified Rankin Scale (mRS).

### Statistical analysis

All data in the graphs are expressed as means ± SD. Patients' backgrounds were compared between the edaravone(-) and edaravone(+) groups using Unpaired Student's *t*-test. The relationship of the alterations of the lesion size ratio to elapsed time was assessed by simple regression analysis. A nonparametric Mann-Whitney's U test was used for the analysis of the difference of the average lesion size ratio between the edaravone(-) and edaravone(+) groups at the same periods.

Nonparametric Kruskal-Wallis test was used for comparison of the difference of mRS distribution between edaravone(-) and edaravone(+) groups. All calculations were performed by the assistance of computer software (Stat View J 5.0. SAS Institute Inc., Cary, NC). Values of p < 0.05 were considered significant.

## Results

### Clinical backgrounds

There were no statistically significant differences of background data, including age, sex and clinical risks between the edaravone(-) and edaravone(+) groups at onset of the ischemic insult (Table 1). Moreover, the distribution of stroke subtypes, the average lesion size and the neurological severity at onset were also not significantly different between the edaravone(-) and edaravone(+) groups (Table 2).

**Table 1:** Patients' backgrounds at onset.

	Edaravone(-) (n = 83)	Edaravone(+) (n = 93)
Female (%)	29 (35)	28 (30)
Age (years old)	69.9 ± 10.67	67.2 ± 10.49
Hypertension (n, %)	48 (57.8)	62 (66.7)
Hyperlipidemia (n, %)	22 (26.5)	34 (36.6)
Diabetes Mellitus (n, %)	23 (27.7)	25 (26.9)
Smoking (n, %)	30 (36.1)	36 (38.7)
Alcohol drinking (n, %)	36 (43.4)	35 (37.6)
Atrial fibrillation (n, %)	18 (21.7)	17 (18.3)

**Table 2:** Clinical data on admission and at discharge in each stroke subtype.

	Edaravone(-) (n = 83)	Edaravone(+) (n = 93)
Cardioembolism (%)	28 (33.7)	21 (22.6)
Average lesion size (mm^2^)	1359.8 ± 1174.1	1391.3 ± 865.9
Hospital duration (days)	39.5 ± 52.1	36.2 ± 21.4
NIHSS on admission①	6.9 ± 6.2	6.6 ± 6.4
at discharge②	4.6 ± 5.6	6.9 ± 10.5
Improvement rate (①-②)	2.8 ± 3.6	-0.1 ± 9.2
Large-artery (%)	30 (36.1)	38 (40.9)
Average lesion size (mm^2^)	251.9 ± 285.6	235.1 ± 136.6
Hospital duration (days)	24.8 ± 15.2	27.6 ± 25.2
NIHSS on admission①	3.8 ± 3.7	4.0 ± 4.6
at discharge②	2.3 ± 2.8	2.4 ± 3.7
Improvement rate (①-②)	1.6 ± 3.4	1.6 ± 3.2
Small-vessel(%)	25 (30.1)	34 (36.6)
Average lesion size (mm^2^)	67.9 ± 37.2	77.6 ± 40.4
Hospital duration (days)	20.8 ± 12.6	21.8 ± 14.4
NIHSS on admission①	2.2 ± 2.2	4.1 ± 4.6
at discharge②	1.4 ± 1.6	2.1 ± 3.2
Improvement rate (①-②)	0.8 ± 1.0	2.0 ± 2.8 ‡

NIHSS: National Institutes of Health Stroke Scale.

‡: p < 0.05

### Alterations of lesion size

**Figure 1:**
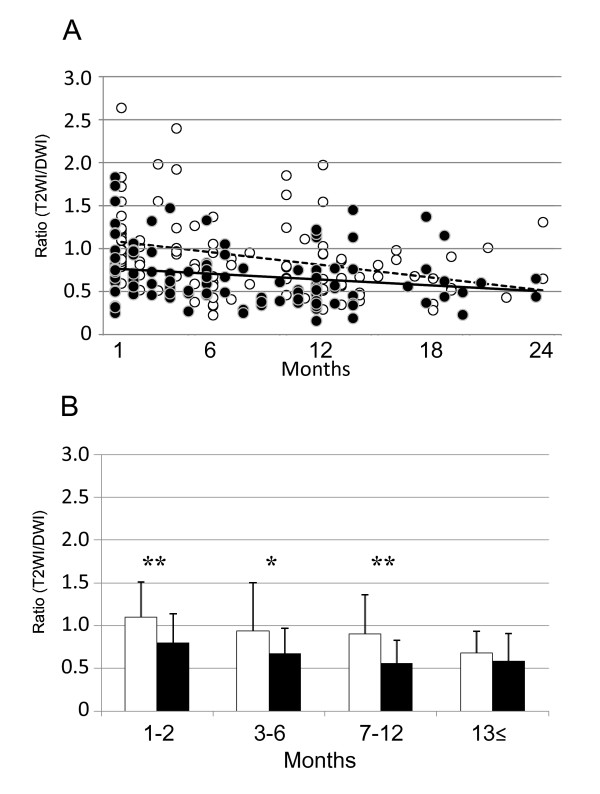
**Alteration of the lesion size**. A: Scatter graphs of the ratios (T2WI/DWI) of edaravone(-) group (open circle) and edaravone(+) group (closed circle). B: The average ratio in edaravone(-) group (white bar) and edaravone(+) group (black bar) in 1-2 months, 3-6 months, 7-12 months and ≥13 months. The regression lines indicate in dotted line as edaravone(-) group (y = 1.10-0.03x, R^2 ^= 0.11, p = 0.001) and in black line as edaravone(+) group (y = 0.78-0.01x, R^2 ^= 0.04, p = 0.031). The average ratio is significantly lower in the edaravone(+) group compared with edaravone(-) group in 1-2 months, 3-6 months and 7-12 months. *: p < 0.05, **: p < 0.01.

The temporal profiles of the stroke lesions were analyzed and plotted (Figure 1). The regression lines indicated that stroke lesions in the edaravone(+) group were reduced at a quicker rate than that in the edaravone(-) group (Figure 1A). The reduction in the size of the stroke lesion was significant in the edaravone(+) group compared with the edaravone(-) group within the first12 months (Figure 1B). When patients were classified into observational times, there was a significant difference between the edaravone(+) and edaravone(-) groups in the subactute (1-2 months; p = 0.006), early chronic (3-6 months; p = 0.012) and late chronic (7-12 months; p = 0.001) periods, but not in the old (**≥**13 months; p > 0.05) period.

**Figure 2:**
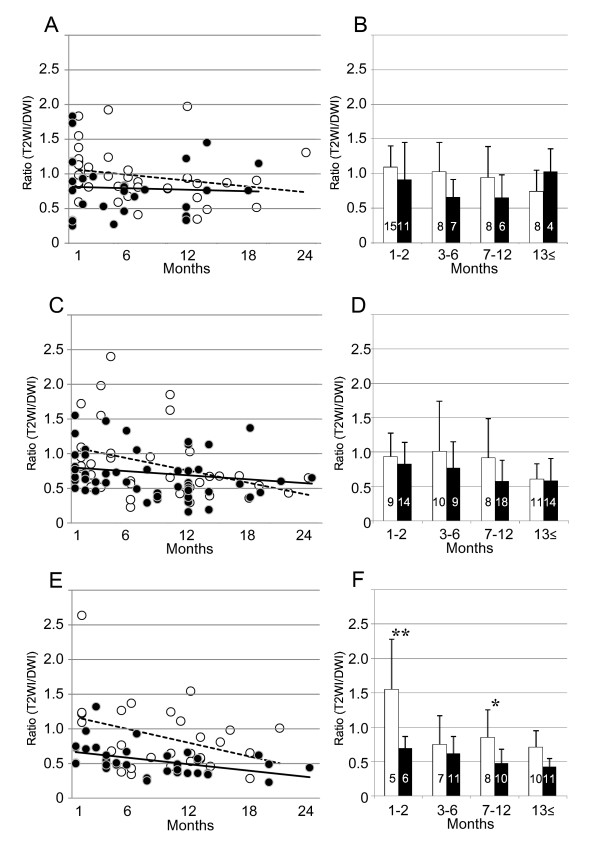
**Alteration of the lesion size by different stroke subtypes**. Scatter graphs of the ratios (T2WI/DWI) of edaravone(-) group (open circle) and edaravone(+) group (closed circle) in cardioembolism (A), large-artery atherosclerosis (C) and small-vessel occlusion (E) stroke subtypes. Average ratios of edaravone(-) group (white bar) and edaravone(+) group (black bar) in the cardioembolism (B), the large-artery atherosclerosis (D) and the small-vessel occlusion (F). Number of cases is indicated in the bar. The regression lines indicate in dotted line as edaravone(-) group and in black line as edaravone(+) group (the formula of cardioembolism: y = 1.07-0.14x, R^2 ^= 0.53, p = 0.160 and y = 0.82-0.004x, R^2 ^= 0.003, p = 0.786, respectively; the formula of large-artery atherosclerosis: y = 1.10-0.03x, R^2 ^= 0.14, p = 0.028 and y = 0.81-0.01x, R^2 ^= 0.03, p = 0.205, respectively; the formula of small-vessel occlusion: y = 1.18-0.32x, R^2 ^= 0.14, p = 0.054 and y = 0.68-0.02x, R^2 ^= 0.20, p = 0.007, respectively). In the small-vessel occlusion, the reduction of lesion size is earlier in edaravone(+) group compared with edaravone(-) group. There are significant differences of the average ratios between edaravone(-) and edaravone(+) groups in 1-2 months and 7-12 months in the small-vessel occlusion. *: p < 0.05 and **: p < 0.01.

Subsequently, we examined the temporal profiles of the ischemic lesions based on stroke subtypes. There were no significant differences between the regression lines or lesion sizes between the edaravone(-) and edaravone(+) groups at any of the observational times for strokes attributed to cardioembolism (Figure 2A, B) or large-artery atherosclerosis (Figure 2C, D). However, the examination of strokes attributed to small-vessel occlusion revealed that edaravone treatment caused a significant reduction in lesions at the subacute (1-2 months; p = 0.001) and late chronic (7-12 months; p = 0.014) periods, but not at the early chronic or old periods (Figure 2E, F).

### ADL assessment

**Figure 3:**
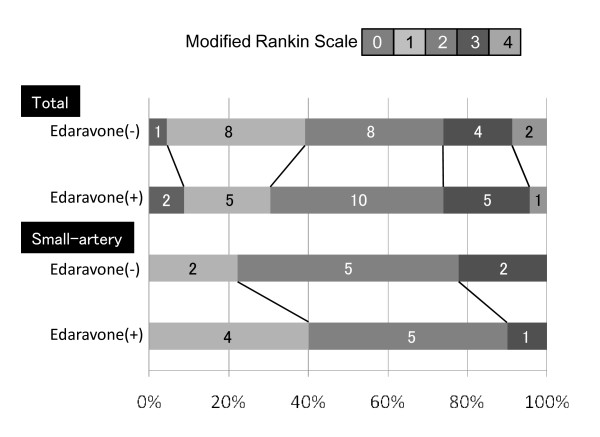
**Neurological outcome**. The distribution of each mRS at one year after the onset. The number of patients is shown in the columns. There is no difference of the distribution of the outcome in total analysis. When only small-vessel occlusion is observed, the percentage of mRS 1 in edaravone(+) group seems increased, although there is no statistical difference between edaravone(-) and edaravone(+) groups.

As shown in Table 2, there was no significant difference in the duration of hospitalization between the edaravone(-) and edaravone(+) groups among different stroke subtypes. In addition, there was no significant difference in neurological deficits at the time of discharge, as assessed by the NIHSS. If the improvement rate was calculated by the difference in the NIHSS scores of each patient (NIHSS on admission - NIHSS at discharge), the edaravone(+) group of the small-vessel occlusion stroke subtype showed significant improvement compared with the edaravone(-) group (p = 0.0465). Moreover, the long-term prognosis (1 year after the stroke) was evaluated using the mRS score. As depicted in Figure 3, there was no significant difference in the mRS distributions between the edaravone(-) and edaravone(+) groups. Even in small-vessel occlusion, which showed a faster reduction of lesion size with edaravone treatment, showed no significant difference in mRS distribution between the edaravone(+) and edaravone(-) groups.

## Discussion

Previous studies have revealed that the free radical scavenger, edaravone, is neuroprotective following acute ischemic stroke in both animal and human models [9,10]. Moreover, the protective effect of edaravone against oxidative stress has been reported to not be limited to neurons, but to also extend to astrocytes and endothelial cells. Thus, edaravone treatment results in protection of the neurovascular unit [11,12]. In the EDO study, edaravone reduced the incidence of recurrent stroke following acute treatment compared with the anti-platelet drug, sodium ozagrel [16]. However, a recent trial with NXY-059, another free radical scavenger, showed no significant neuroprotection when administered to stroke patients (SAINT II) [17]. Collectively, these findings suggest that edaravone may have efficacy in ischemic stroke treatment, but with limitations in its therapeutic capability [18].

In our study, patients treated with edaravone exhibited a significant reduction in ischemic lesion size compared with those not treated with edaravone. However, this finding was limited to specific time frames, or the subacute, early chronic and late chronic phases. Generally, edaravone is administrated twice a day for 2 weeks from the onset of the ischemic insult. Because of its ability to restrict inflammation, edaravone may potentially limit the ischemic insult during the early phases and, thus, restrict the expansion of the stroke lesion faster than those not treated with the free radical scavenger. In support of this finding, edaravone has been reported to reduce delayed neuronal death after middle cerebral artery occlusion by limiting N-acetyl aspartate signaling in humans [19]. Further supporting our findings, brain edema following stroke with internal carotid artery stenosis has also been reported to be limited to the acute period if patients were treated with edaravone [20].

Upon closer examination of our results, lesion reduction elicited by edaravone was most effective in the small-vessel occlusion stroke subtype. In this study, lesions due to small-vessel occlusion were mostly located in corona radiata (white matter) and some were in basal ganglia (gray matter). Anatomically, white matter contains more astrocytes and microglia which participate in the inflammatory response, and more phospholipids which are vulnerable to oxidative stress [21]. Therefore, it is reasonable that edaravone treatment excerted a significant effect on the small-vessel occlusion subtype due to its actions on inflammation. Actually, a recent study reported that edaravone reduced the extent of damage in both the gray and white matter caused by global ischemia [22], supporting the theory that edaravone may act by limiting inflammation.

While effective in the small-vessel occlusion stroke subtype, edaravone failed to elicit statistically significant reductions in lesion sizes in the cardioembolism and the large-artery atherosclerosis subtype groups in our study. It is possible that reductions of lesional expansion by edaravone may be limited by the hemodynamic worsening, which occurs in these specific stroke subtypes. Moreover, because our data included patients with both mild and severe neurological deficits, the effects of edaravone may be masked by its inability to treat ischemic insults unconditionally. In support of this notion, it has been reported that edaravone improves the outcome of embolic strokes only in patients with mild neurological deficits [23]. Furthermore, edaravone treatment appeared to trend towards lesion size reductions in all subtypes, and not just the small-vessel occlusion stroke subtype, suggesting that the small sample sizes in this study may have prevented the identification of statistical significance between the treatment groups. This was overcome when the stroke subtypes were grouped collectively.

Our findings indicate that treatment with edaravone did not cause any significant differences in clinical outcomes, such as the NIHSS at discharge point, duration of hospital days, and the mRS at one year after the onset. However, a significant improvement in clinical outcome at discharge was observed in the small-vessel occlusion stroke subtype treated with edaravone. These findings are supported by a recent study on acute lacunar infarctions in which Ohta et al. found that a protective effect of edaravone was not identified using NIHSS scores but could be identified when using palsy scores [24].

A limitation of our study is that we examined a retrospective cohort that was gathered from a two year period, and the patients were consecutively treated according to the typical treatment regimens with and without edaravone, as described in the methods section. A fully-powered, prospective, randomized control trial may expand on our findings and demonstrate the full benefits of edaravone in patients with ischemic insults. Indeed, current clinical trials are being conducted with free radical scavengers, including a study of the safety and pharmacokinetics of MCI-186 in subjects with acute ischemic stroke in Europe [25].

## Conclusion

Edaravone can effectively reduce the size of ischemic stroke lesions and improve neurological deficits in patients with small-vessel occlusion, i.e., lacunar infarction. Further studies are needed to identify the true benefits and limitations of this free radical scavenger in cerebral ischemia.

## Competing interests

The authors declare that they have no competing interests.

## Authors' contributions

TN conducted this study and performed the statistical analysis. SY carried out the screening of patients' data and performed the measurement of lesion sizes. AS participated in setting up and coordination of this study. All authors read and approved the final manuscript.

## Pre-publication history

The pre-publication history for this paper can be accessed here:

http://www.biomedcentral.com/1471-2377/11/39/prepub
